# Cancer in multiple sclerosis patients following prolonged exposure to disease-modifying therapies (DMTs): a systematic review and meta-analysis

**DOI:** 10.1007/s00415-024-12882-4

**Published:** 2025-01-23

**Authors:** Vasileios Giannopapas, Vassiliki Smyrni, Dimitrios K. Kitsos, Maria Ioanna Stefanou, Aikaterini Theodorou, John S. Tzartos, Georgios Tsivgoulis, Sotirios Giannopoulos

**Affiliations:** 1 Second Department of Neurology, Attikon University Hospital, National & Kapodistrian University of Athens, Athens, Greece; 2 Department of Physical Therapy, University of West Attica, Athens, Greece

**Keywords:** Multiple sclerosis, Autoimmune diseases, Cancer, Malignancy, Disease-modifying therapies

## Abstract

**Introduction:**

The current literature on the prevalence and potential association between disease-modifying therapies (DMTs) and cancer risk in the MS population has yielded mixed findings.

**Methods:**

This study aimed to estimate cancer prevalence and cancer risk in patients with MS (PwMS) under prolonged DMT exposure. Database search include: MEDLINE PUBMED, SCOPUS, and Google Scholar.

**Results:**

A total of 13 studies involving 333,779 PwMS were included, reporting cancer events over periods ranging from 6 to 32 years. The aggregated pooled prevalence of cancer events in MS patients receiving disease-modifying therapies (DMTs) was 3.8% (95% CI 2.6, 5.2%), with substantial heterogeneity (*I*^2^ = 99.7%, *p* = 0). Two studies compared cancer events in MS patients who received DMTs versus those who did not. The relative risk of cancer associated with DMTs was 0.8 (95% CI 0.59–1.31, *I*^2^ = 93.6%, *p* = 0.53), indicating no significant increase in cancer risk due to DMTs. Breast and basal cell carcinomas had a high prevalence (18.4% and 11.3, respectively) in PwMS under DMTs.

**Conclusion:**

This study reports a 3.8% pooled prevalence of cancer in PwMS receiving DMTs. The findings of this study suggest that DMTs alone do not increase cancer risk in PwMS. Breast cancer and basal cell carcinoma had the highest prevalence among the different types of cancer.

**Supplementary Information:**

The online version contains supplementary material available at 10.1007/s00415-024-12882-4.

## Introduction

Multiple sclerosis (MS) is a chronic autoimmune inflammatory disease characterized by loss of myelin in the central nervous system [1]. Disease-modifying therapies (DMTs) are employed to curb disease activity, slow the progression of physical disability associated with the disease, and improve its clinical course [2]. DMTs are usually initiated shortly after MS diagnosis, raising the need for accurate evaluation of the long-term health-related implications of these treatments [3].

Although the link between autoimmune diseases and cancer is incompletely understood, several researchers have suggested a potential link between MS treatments and elevated cancer risk [4–7]. According to the current literature, the pathogenesis of MS is thought to involve an improper immune response, which is driven by autoreactive lymphocytes within the central nervous system [1]. Several studies have reported that prolonged exposure to DMTs could induce immunological response alterations, affecting T cell activation, which may potentially increase the cancer risk in patients with MS (PwMS) [4–8]. Conversely, a number of studies have not been able to establish a link between DMTs and cancer occurrence in the MS population, on the basis that autoreactive T cells can also play a part in cancer prevention [9, 10].

Given the contradictory findings in the current literature and the potential influence of DMTs on cancer risk, it is essential to evaluate their long-term effects beyond the scope of short-duration clinical trials. Hence, this systematic review and meta-analysis aims at estimating the prevalence of cancer in PwMS under prolonged DMT exposure.

## Methods

### Standard protocol approvals, registrations

The pre-specified protocol of this systematic review and meta-analysis is registered in the Open Search Foundation (OSF) (Registration: osf.io/nwdyb). The meta-analysis was reported as per the updated Preferred Reporting Items for Systematic Reviews and Meta-Analyses (PRISMA) guidelines [11] and was written in accordance with the Meta-analysis of Observational Studies in Epidemiology (MOOSE) proposal [12]. The study did not require an ethical board approval or written informed consent.

### Data sources, searches and study selection

A systematic literature search was conducted to identify eligible studies reporting on cancer events in PwMS under DMTs. The literature search was performed by two independent reviewers (VG, VS). The MEDLINE, PubMed, and Scopus databases were searched, as well as the first 200 results of Google Scholar. A systematic literature search was conducted to identify eligible studies reporting on cancer events in PwMS who had or had not received DMTs that was performed independently by two reviewers (VG, VS). Search queries included the terms: “multiples sclerosis”, “MS”, “cancer”, “cancer incidence”, “malignancy”, “neoplasm”, “DMTs”, “disease-modifying therapies”, and “disease-modifying drugs”. No language or other restrictions were applied. Our search spanned from inception of each electronic database to July 20^th^, 2024.

Per study protocol, we excluded studies: (1) including patients with diagnosis of clinically isolated syndrome (CIS); (2) uncertain MS and/or cancer diagnosis; (3) reporting outcomes not aligned with our inclusion criteria; (4) clinical trials, case series, case reports, commentaries, narrative and systematic reviews, non-peer reviewed studies, pre-prints and conference abstracts. In the case of overlapping data between studies, the study with the largest dataset was retained. All retrieved studies were independently assessed by two reviewers (VG, VS), and any disagreements were resolved after discussion with a third tie-breaking evaluator (SG).

### Quality control, bias assessment and data extraction

The risk of bias for relevant domains in each included study was assessed by two reviewers (VG, VS), using the Risk of Bias In Non-randomized Studies of Interventions (ROBINS-I) [13]. Any disagreements were settled by the corresponding author (SG). Data from individual studies, including author names, date of publication, study design, country, and events, were extracted.

### Outcomes and statistical analysis

An aggregate data meta-analysis was performed which included the identified population-based studies or registries, and observational cohort studies.

The predefined primary outcome measures were twofold: (i) the pooled prevalence of cancer in PwMS under DMTs; (ii) the pooled relative risk of cancer; and (iii) the pooled prevalence of different cancer types in PwMS under DMTs.

### Statistical analysis

For the aggregate meta-analysis, the pooled prevalence with the corresponding 95% confidence interval (95% CI) was calculated by the R-meta meta-prop function using the Freeman–Tukey variance-stabilizing double arcsine transformation and the random-effects model for the calculation of pooled estimates (DerSimonian and Laird) [14]. Heterogeneity was assessed with the *I*^2^ index and Cochran Q statistics [15, 16]. The significance level for the Q statistic was set at 0.1 in order to detect statistical trends. Publication bias across individual studies was assessed by Funnel plot inspection and the result of the Egger’s linear regression test [17]. All statistical analyses and figure production were carried out using RStudio for IOS [R studio/R Meta package] [11].

### Data availability statement

All data generated or analyzed during this study are included in this article and its supplementary information files.

## Results

### Literature search

**Fig. 1:**
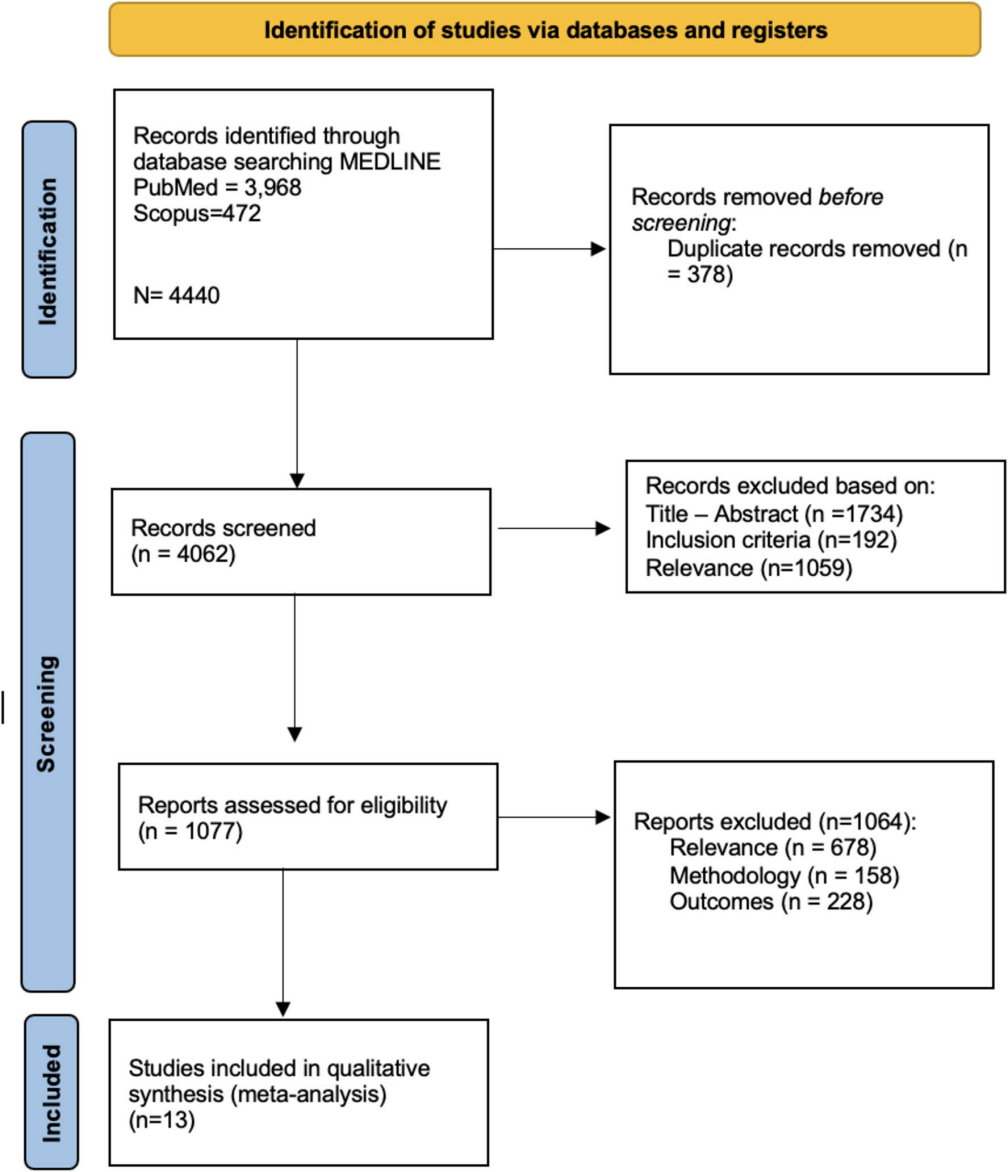
PRISMA flow chart

The systematic literature review yielded a total of 4440 results. After duplicate extraction, removal of systematic reviews and meta-analysis, removal of studies that did not include PwMS and implementation of selection criteria 13 studies were included (Fig. 1).

### Quality and risk of bias assessment

**Fig. 2:**
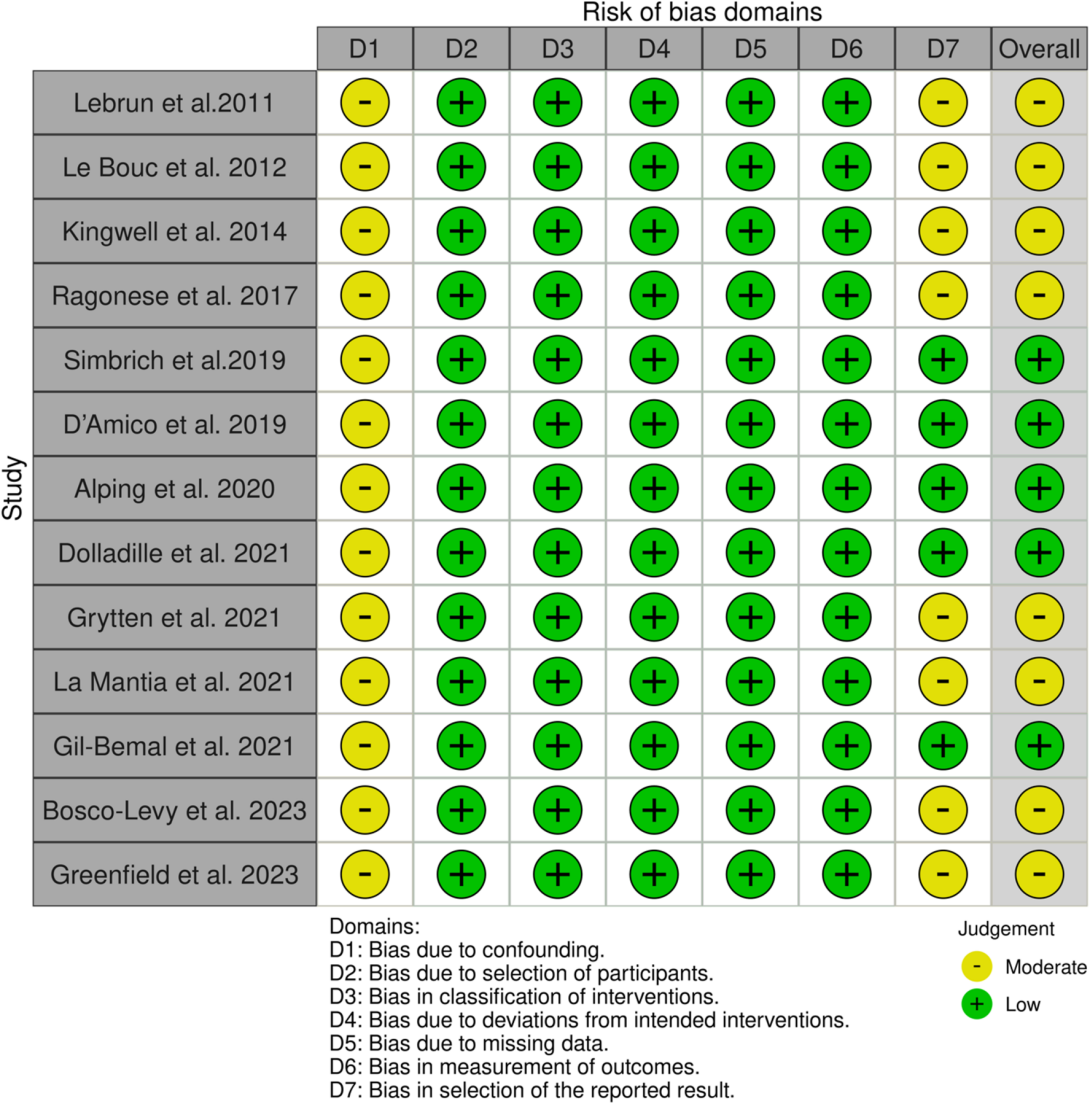
ROBINS-I traffic light plot

**Fig. 3:**
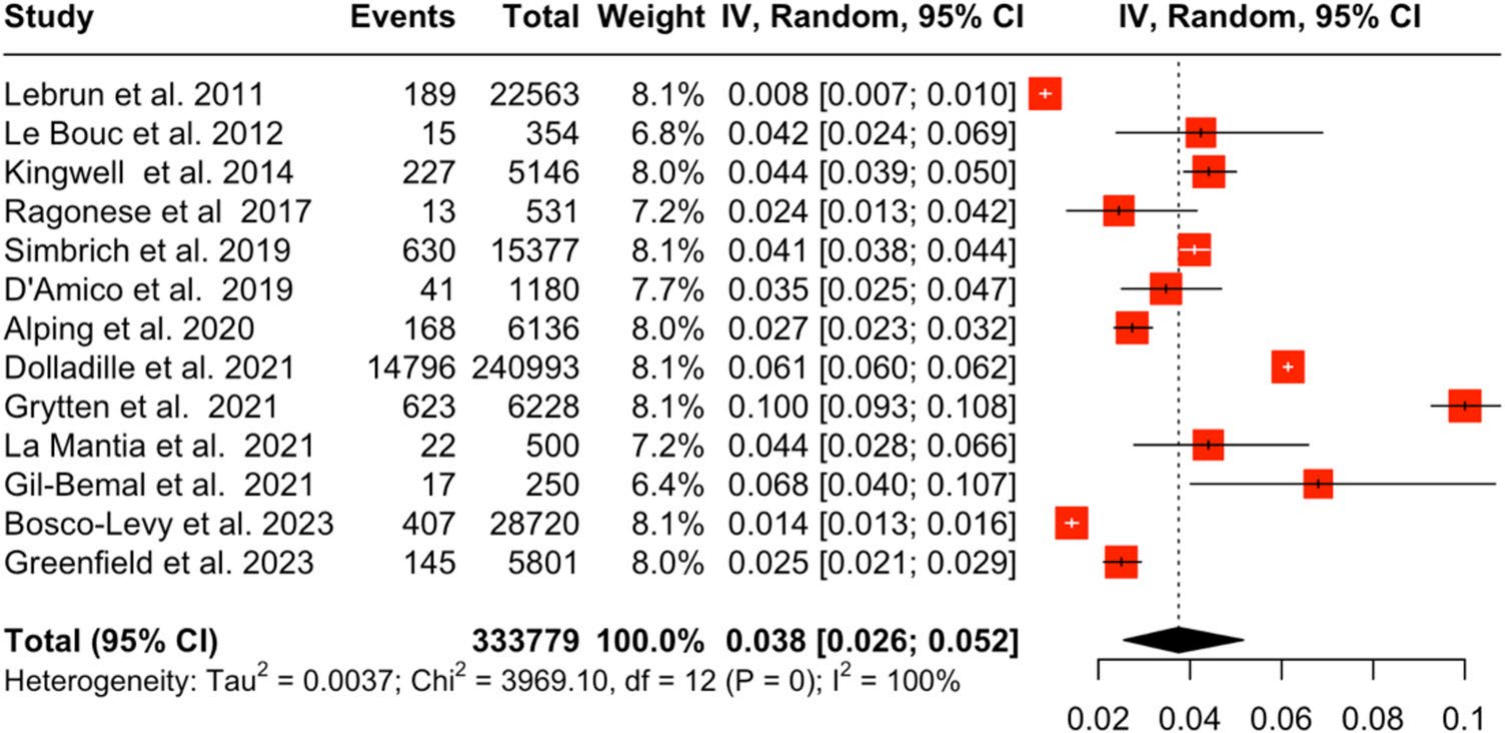
Forest plot of pooled cancer prevalence in PwMS under DMTs versus PwMS not receiving DMTs

**Fig. 4:**
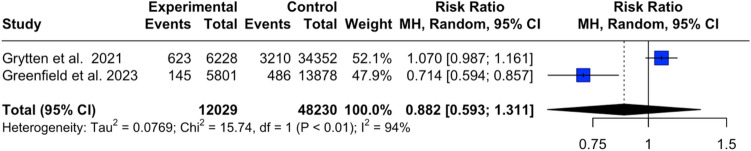
Forest plot: relative risk of cancer in PwMS under DMTs

Included studies were assessed using the ROBINS-I with most of them reporting moderate bias in the confounding variables and reported outcomes domains (Figs. 2, 3, 4).

### Quantitative results

**Fig. 5:**
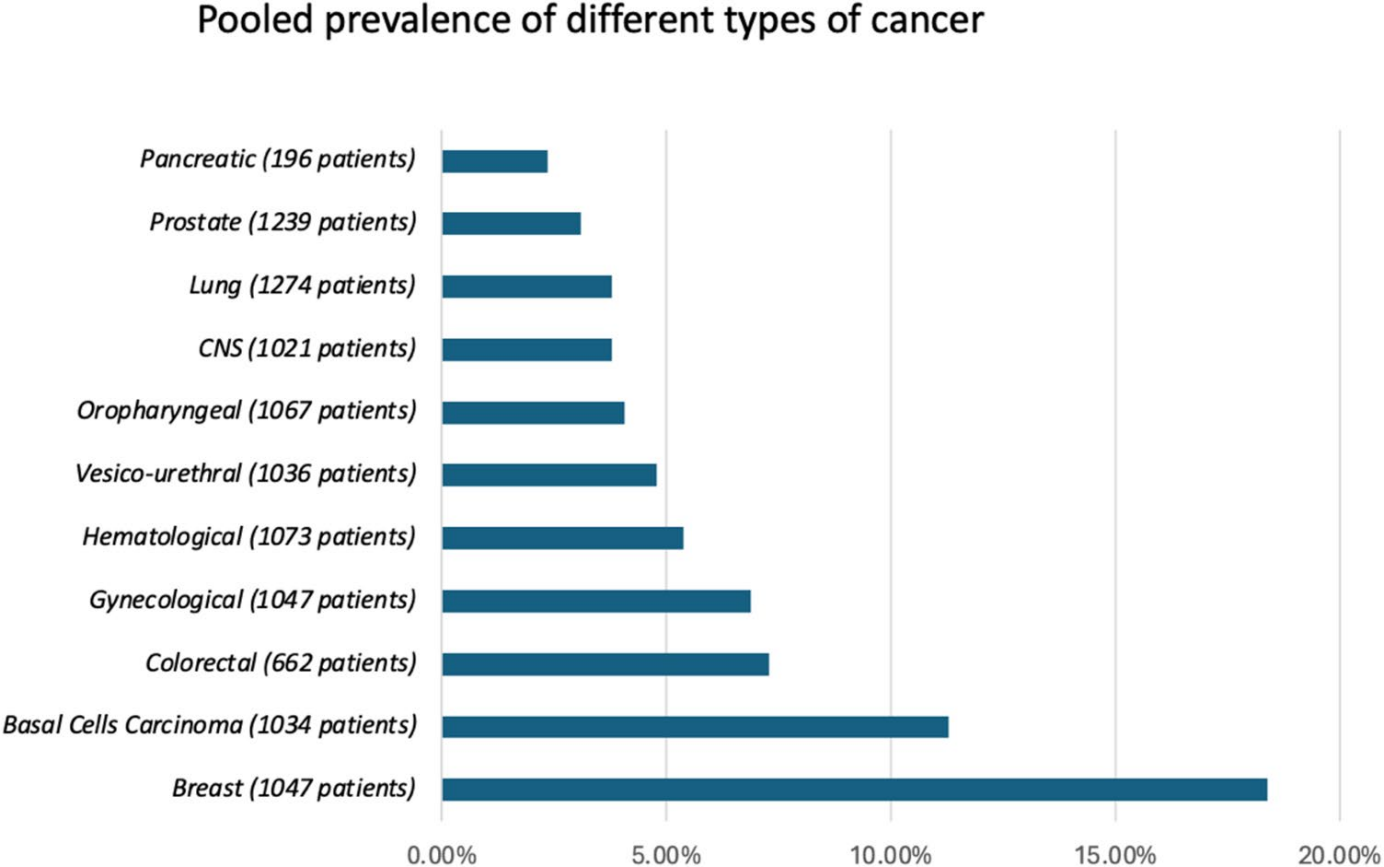
Pooled prevalence of different types of cancer in PwMS under DMTs

13 studies [3–5, 18–27] with 333,779 PwMS were included. Studies reported data on cancer events over a period of 6–32 years (Table 1)**.** The aggregated pooled prevalence of cancer events in PwMS receiving DMTs was 3.8% (95% CI [2.56, 5.16], *I*^2^ = 99.7%, *p*_Q_ = 0) (Fig. 3). Two studies reported cancer events in MS patients, which compared individuals who had received DMTs to those who had not. The relative risk of cancer due to DMTs was 0.88 (95% CI [0.59, 1.31], *I*^2^ = 93.6%, *p*_Q_ < 0.001, *p*_z_ = 0.53) (Fig. 4) indicating that DMTs do not increase cancer risk. Subsequent subset analyses regarding the prevalence of different types of cancers in PwMS were performed and are presented in Table 2 (Fig. 5, and Supplementary Figs. S1–S10).

**Table 1:** Included studies

Author	Sample	Cancer events	Disease duration	Age at cancer diagnosis (years)	EDSS
Lebrun et al. 2011 [27]	22,563	182	–	48.1	–
Le Bouc et al. 2012 [18]	354	15	–	59.8	–
Kingwell et al. 2014 [19]	5146	227	24.2	60.9	–
Ragonese et al. 2017 [20]	531	13	19.2	–	–
Simbrich et al. 2019 [21]	15,377	630	4.5	–	–
D’Amico et al. 2019 [22]	1180	36	10	49.3	4.2
Alping et al. 2020 [23]	6136	168	8.85	39.2	2.5
Dolladille et al. 2021 [4]	240.993	5.966	19	–	–
Grytten et al. 2021 [24]	34,352	6228	-	50.4	–
La Mantia et al. 2021 [5]	500	22	21.7	–	3.7
Gil-Bemal et al. 2021 [25]	250	17	38	39.8	–
Bosco-Levy et al. 2023 [26]	28,720	407	6	–	–
Greenfield et al. 2023 [3]	5801	145	8.4	–	–

**Table 2:** Pooled prevalence of different types of cancer

Cancer type	Pooled sample size	Prevalence (%, CI)
Pancreatic	196	2.3% [0, 10.6]
Prostate	1239	3.1% [0, 6.1]
Lung	1274	3.8% [0.8, 7.5]
CNS	1021	3.8% [0, 8]
Oropharyngeal	1067	4.0% [0.1, 11.2]
Vesico-urethral	1036	4.8% [1.3, 9.9]
Hematological	1073	5.4% [1.3, 11.4]
Gynecological	1047	6.9% [2.7, 12.5]
Colorectal	662	7.3% [5.6, 10.0]
Basal cell carcinoma	1034	11.3% [4.3, 20.7]
Breast	1046	18.4% [7.6, 32.0]

Finally, in a set of 10 studies that reported data on MS disease duration, meta-regression revealed a statistically significant association between MS disease duration and cancer prevalence (*β* = 0.003, *p* = 0.01) which translates to 0.3% increase in cancer prevalence every 1-year post-MS diagnosis. There was no statistically significant relationship between cancer prevalence and age at cancer diagnosis (*β* = 0.0006, *p* = 0.87) or cancer prevalence and EDSS (*β* = 0.01, *p* = 0.23).

### Publication bias

Publication bias was assessed for studies reporting cancer events. Funnel plot inspection revealed asymmetries with a non-significant Egger’s linear regression test (*β* = −8.35, *p* = 0.22) which are indicative of moderate publication bias (Supplementary Fig. 12).

## Discussion

This SR-MA included 13 studies with a total of 333,779 PwMS receiving DMTs, with a pooled cancer prevalence of 3.8%. Included studies reported non-stratified data for cancer events in PwMS under different DMTs that included Interferon beta, acetate, Dimethyl fumarate, Teriflunomide, Fingolimod, Natalizumab, Alemtuzumab, Ocrelizumab, Rituximab, Azathioprine, Mitoxantrone, Methotrexate, and Cyclophosphamide. The findings of this study are in close proximity to the percentage yielded by Marrie et al., who reported 4.39% prevalence of any type of cancer in PwMS [28]. Compared to the data from the last GLOBACAN report where cancer incidence ranged from 0.1 to 12.4% (based on cancer type and location), the pooled cancer prevalence in PwMS produced by our study places the prevalence of cancer in PwMS under DMT exposure in the 8^th^ place (out of the 33), while breast cancer and basal cell carcinomas in PwMS under prolonged DMT exposure are placed even higher [29]. Moreover, in the subsequent subset analysis of studies that included MS patients with and without DMTs, the pooled risk ratio of cancer under DMT exposure was estimated to be 0.81 (95% CI [0.59, 1.31]), indicating that exposure to DMTs does not increase the risk of cancer development. It is worth noting that based on the limited data regarding MS specific characteristics, disease duration has a statistically significant association with cancer prevalence (0.3% increase every year), whereas disability level (as measured by the EDSS) has no significant association with cancer prevalence.

With regards to the prevalence of the various cancer types among patients under DMTs, our findings are in accordance to previous studies which have reported an elevated risk of breast and skin cancers in PwMS and is correlated to exposure to either ocrelizumab or S1P receptor modulators [30, 31]^.^ Especially in the case of breast cancer in MS, these findings are not surprising since breast cancer and MS are both female-dominant conditions. Nevertheless, it is worth noting that although both conditions are female dominant, previous research which analyzed data from observational and Mendelian randomization studies has found no statistically significant correlation between specific genetic variants for breast cancer and MS [32].

On the other hand, several studies have explored the proposed anti-cancer properties of different DMTs. Sphingosine 1-Phosphate Receptor (S1PR) modulators such as Siponimod and fingolimod have been shown to inhibit sphingosine kinase 1 (SK1). This enzyme is upregulated in various forms cancers, including CNS, gastrointestinal tract, genitourinary system, lungs, and breast cancers [33, 34]. Previous studies have reported a statistically significant association between long-term use of S1P receptor modulators and the development of basal cell carcinoma and melanoma, suggesting a heightened risk of skin cancers in the MS population. This could be potentially attributed to safety signaling and reduction of circulating lymphocytes which are responsible for the identification and elimination of malignant cells [31]. On the contrary, recent evidence has demonstrated the anti-cancer properties of anti-CD20 B-cell therapies (including rituximab, ocrelizumab, and ofatumumab) against B-cell lymphomas. The main hypothesis poses that anti-CD20 B-cell therapies target the CD20 cell surface marker on malignant B cells. Lastly, despite the aforementioned findings, it is important to also acknowledge the effect of the interference of B-cell therapies with the tumor’s microenvironment, which could explain the observed cancer predisposition among patients receiving B-cell therapies [35, 36].

Finally, the anti-cancer role of DNA synthesis inhibitors (DSIs), such as Dimethyl Fumarate, should not be overlooked, as they reduce inflammatory T cells via the nuclear factor-erythroid 2-related factor 2(Nrf2) and the upregulation of glutathione involved in the antioxidant response [37]. Additional evidence suggests that DSIs may downregulate the Nrf2-DJ-1 antioxidant pathway, subsequently causing cancer cell induction. More specifically, in several patients with melanoma treated with DSIs, they were shown to inhibit cell proliferation, induce apoptosis and arrest of the cell cycle, consequently reducing the cancer risk in these patients [37].

It is worth noting that even though DMTs do not seem to increase the risk of cancer in PwMS, research poses that escalating or de-escalating by switching DMTs can lead to higher cancer risk [22].

### Limitations

This study is not without its limitations. To begin with, there is increased heterogeneity between the included studies, with moderate evidence of publication bias. Second, most of the studies did not provide data on the participants’ age and disability level (EDSS) at the time of cancer diagnosis, thus not allowing for sensitivity analysis to be performed, which would allow us to examine of the role of neuroinflammation and/or neurodegeneration in cancer prevalence. Third, the majority of the included studies did not report the number of DMT switches and the corresponding cancer events, nor did they report the duration of exposure for every included DMT which prevented us from performing sensitivity analyses to further assess the role of DMT exposure in cancer prevalence.

## Conclusions

This study found a 3.8% pooled prevalence of cancer in PwMS receiving DMTs. The findings of this study suggest that DMTs alone do not increase cancer risk in PwMS. However, this study found that among the different types of cancer seen in PwMS, there is an increased prevalence of breast cancer and basal cell carcinoma in these patients, which may be associated with specific prolonged DMT exposure. These observations are further supported by previous studies which have suggested that switching DMTs may be linked to higher cancer prevalence in PwMS. This study highlights the need for individualized treatment decisions, balancing benefits against potential cancer risks, patients’ education and systematic testing. Studies that systematically record DMT exposure (including type of DMT, duration of use and number of switches) in PwMS who later develop cancer are crucial to advance our understanding on the potential cancer risk resulting from DMT use.

## Supplementary Information

Below is the link to the electronic supplementary material.

Supplementary file1 (DOCX 2287 KB)

Supplementary file2 (DOCX 32 KB)

## Data Availability

Data are available from the corresponding author upon reasonable request.
