# Supplementary material for: Cancer in multiple sclerosis patients following prolonged exposure to disease-modifying therapies (DMTs): a systematic review and meta-analysis
Source: J Neurol. 2025 Jan 23;272(2):162. doi: 10.1007/s00415-024-12882-4 (PMC11757934; doi:10.1007/s00415-024-12882-4)
Supplement: Supplementary file 1 — Supplementary file1 (DOCX 2287 KB) [file 415_2024_12882_MOESM1_ESM.docx]

**Supplementary material**

1. **Search Algorithms**

PUBMED- ((multiple sclerosis) AND ((cancer) OR (Disease Modifying Therapies) OR (Disease Modifying Drugs))

PUBMED- ((multiple sclerosis) AND ((malignancy) OR (Disease Modifying Therapies) OR (Disease Modifying Drugs))

PUBMED- ((multiple sclerosis) AND ((neoplasm) OR (Disease Modifying Therapies) OR (Disease Modifying Drugs))

Scopus- ((multiple sclerosis) AND ((cancer) OR (Disease Modifying Therapies) OR (Disease Modifying Drugs))

Scopus- ((multiple sclerosis) AND ((malignancy) OR (Disease Modifying Therapies) OR (Disease Modifying Drugs))

Scopus- ((multiple sclerosis) AND ((neoplasm) OR (Disease Modifying Therapies) OR (Disease Modifying Drugs))

1. **Supplementary Figures**

**Supplementary Figure 1: Forest plot: Pooled prevalence of Pancreatic cancer in PwMS under DMTs**

**Supplementary Figure 2: Forest plot: Pooled prevalence of Prostate cancer in PwMS under DMTs**

**Supplementary Figure 3: Forest plot: Pooled prevalence of Lung cancer in PwMS under DMTs**

**Supplementary Figure 4: Forest plot: Pooled prevalence of CNS cancer in PwMS under DMTs**

**Supplementary Figure 5: Forest plot: Pooled prevalence of Oropharyngeal cancer in PwMS under DMTs**

**Supplementary Figure 6: Forest plot: Pooled prevalence of Vesico-urethral cancer in PwMS under DMTs**

**Supplementary Figure 7: Forest plot: Pooled prevalence of Hematological cancer in PwMS under DMTs**

**Supplementary Figure 8: Forest plot: Pooled prevalence of Gynecological cancer in PwMS under DMTs**

**Supplementary Figure 9: Forest plot: Pooled prevalence of Colorectal cancer in PwMS under DMTs**

**Supplementary Figure 10: Forest plot: Pooled prevalence of Basal Cells Carcinomas in PwMS under DMTs**

**Supplementary Figure 11: Forest plot: Pooled prevalence of Breast cancer in PwMS under DMTs**

**Supplementary Figure 12: Funnel plot for publication bias**

**Supplementary Figure 13: Prisma Checklist**

**Supplementary Figure 1: Forest plot: Pooled prevalence of Pancreatic cancer in PwMS under DMTs**

**
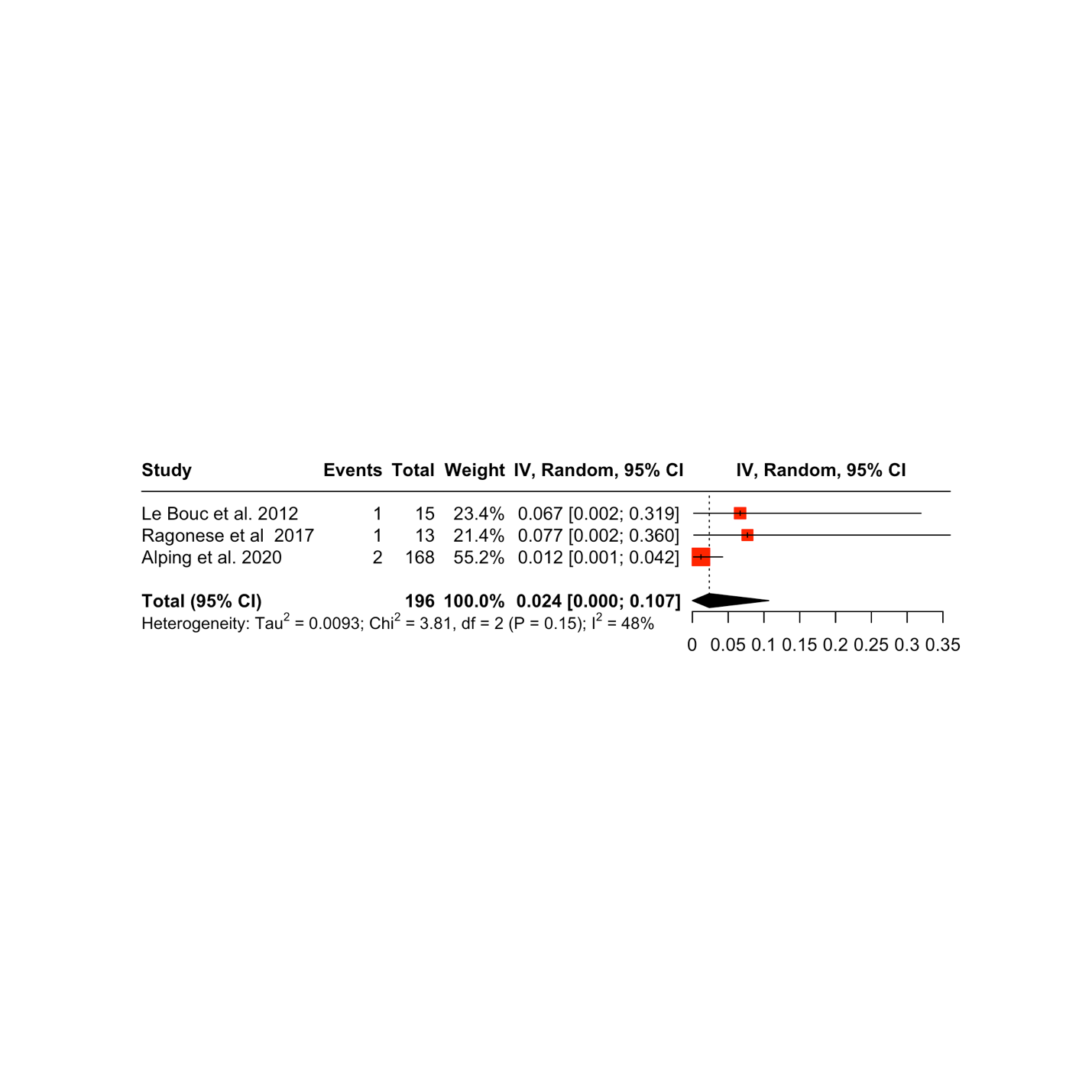
**

**Supplementary Figure 1: Forest plot: Pooled prevalence of Prostate cancer in PwMS under DMTs
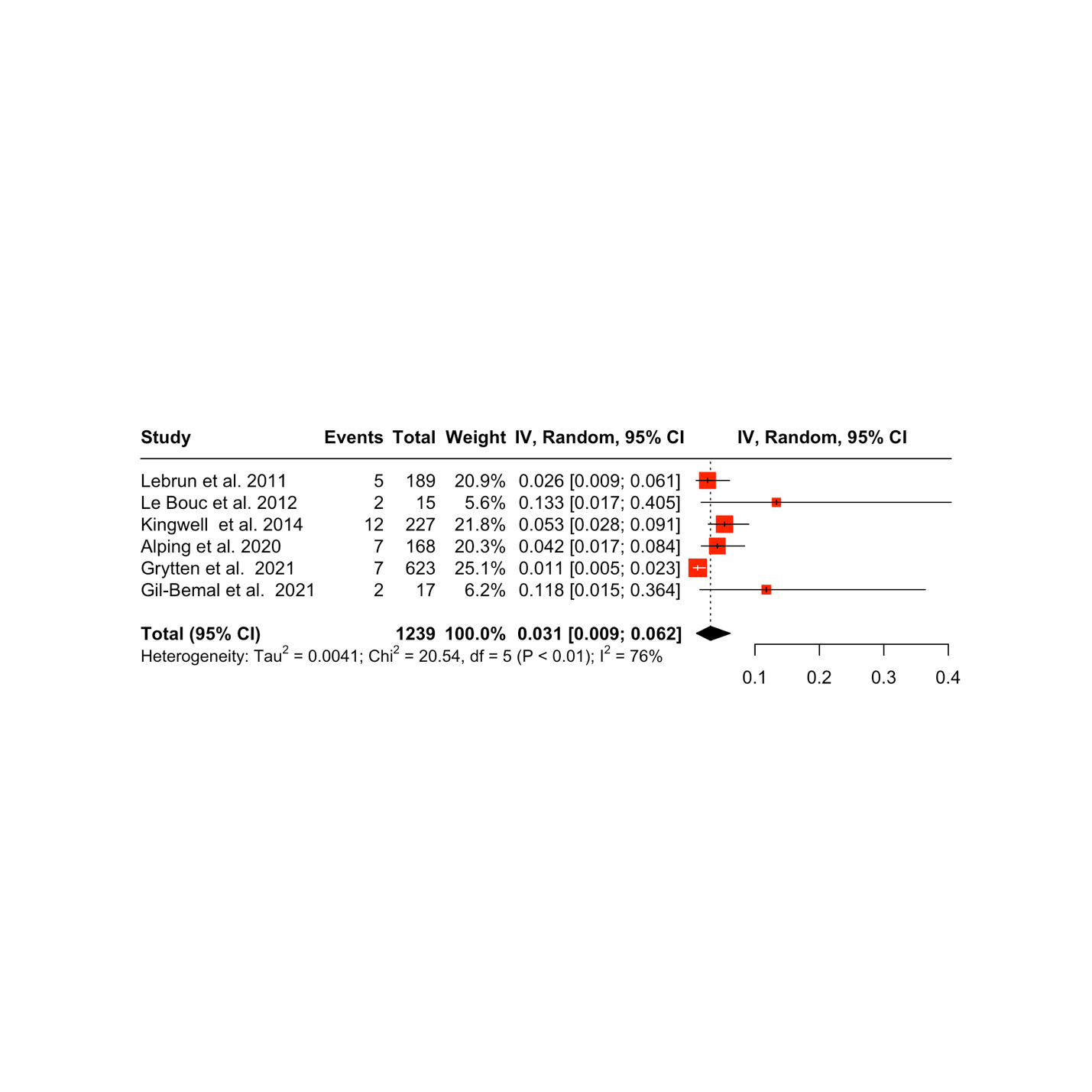
**

**Supplementary Figure 3: Forest plot: Pooled prevalence of Lung cancer in PwMS under DMTs**

**
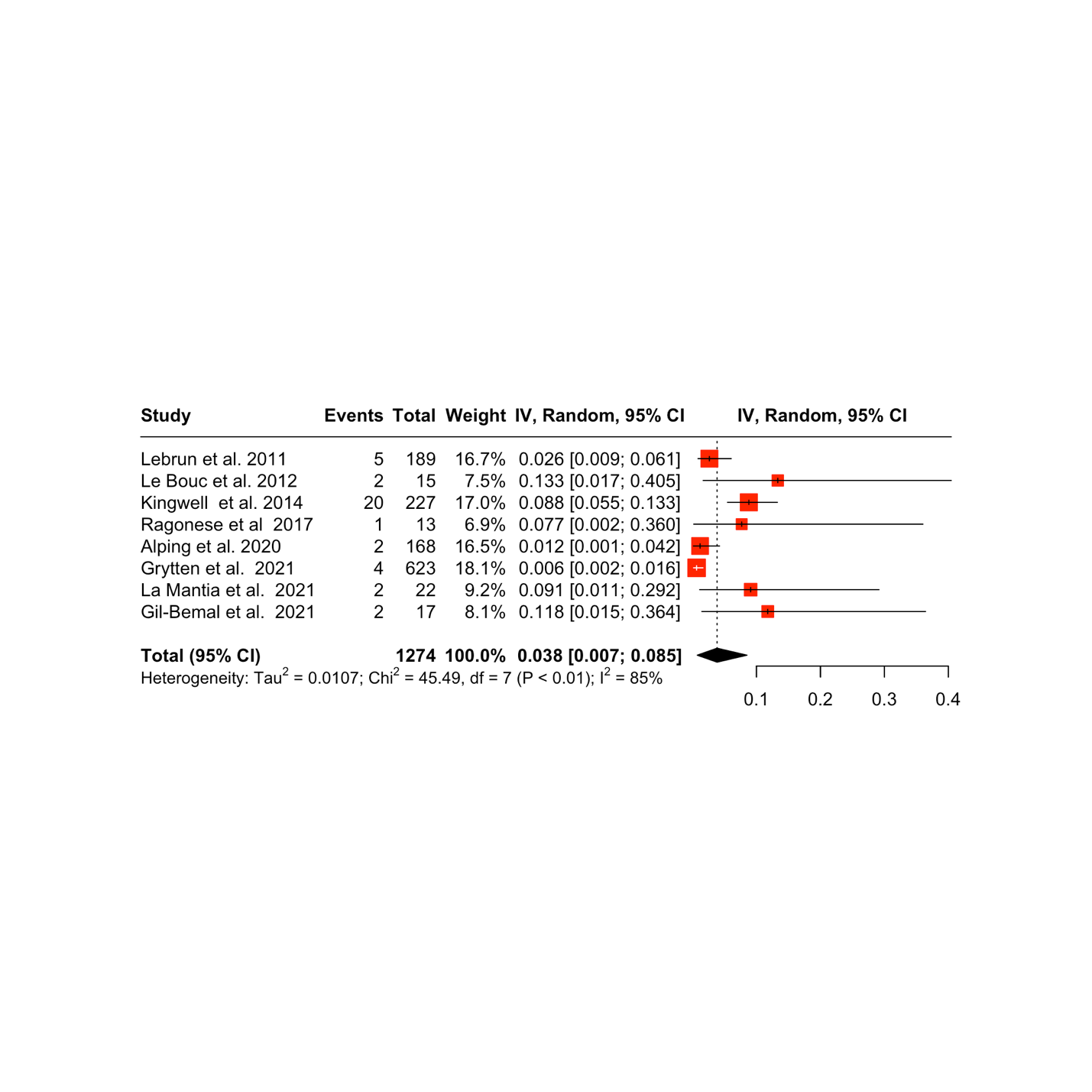
**

**Supplementary Figure 4: Forest plot: Pooled prevalence of CNS cancer in PwMS under DMTs**

**
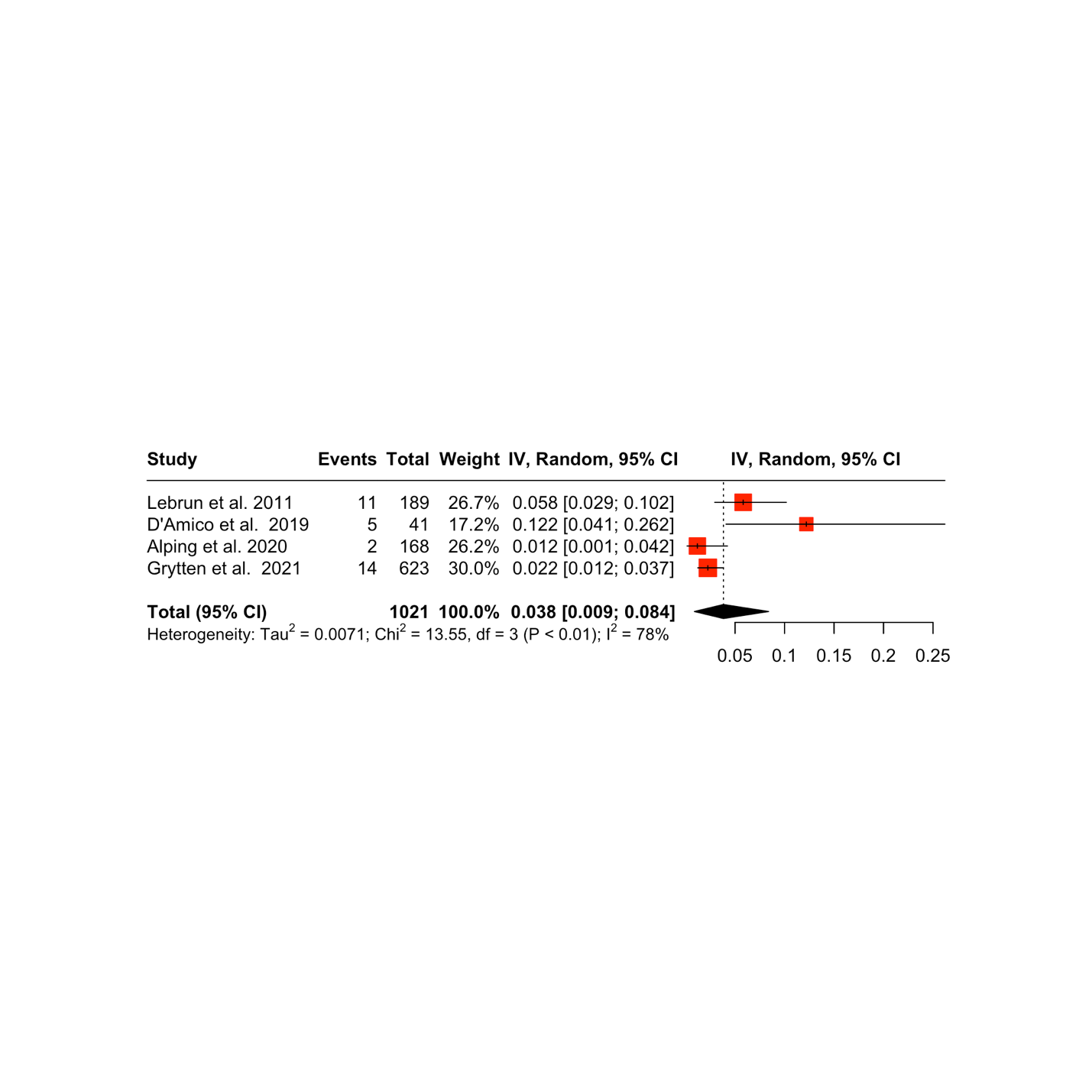
**

**Supplementary Figure 5: Forest plot: Pooled prevalence of Oropharyngeal cancer in PwMS under DMTs**

**
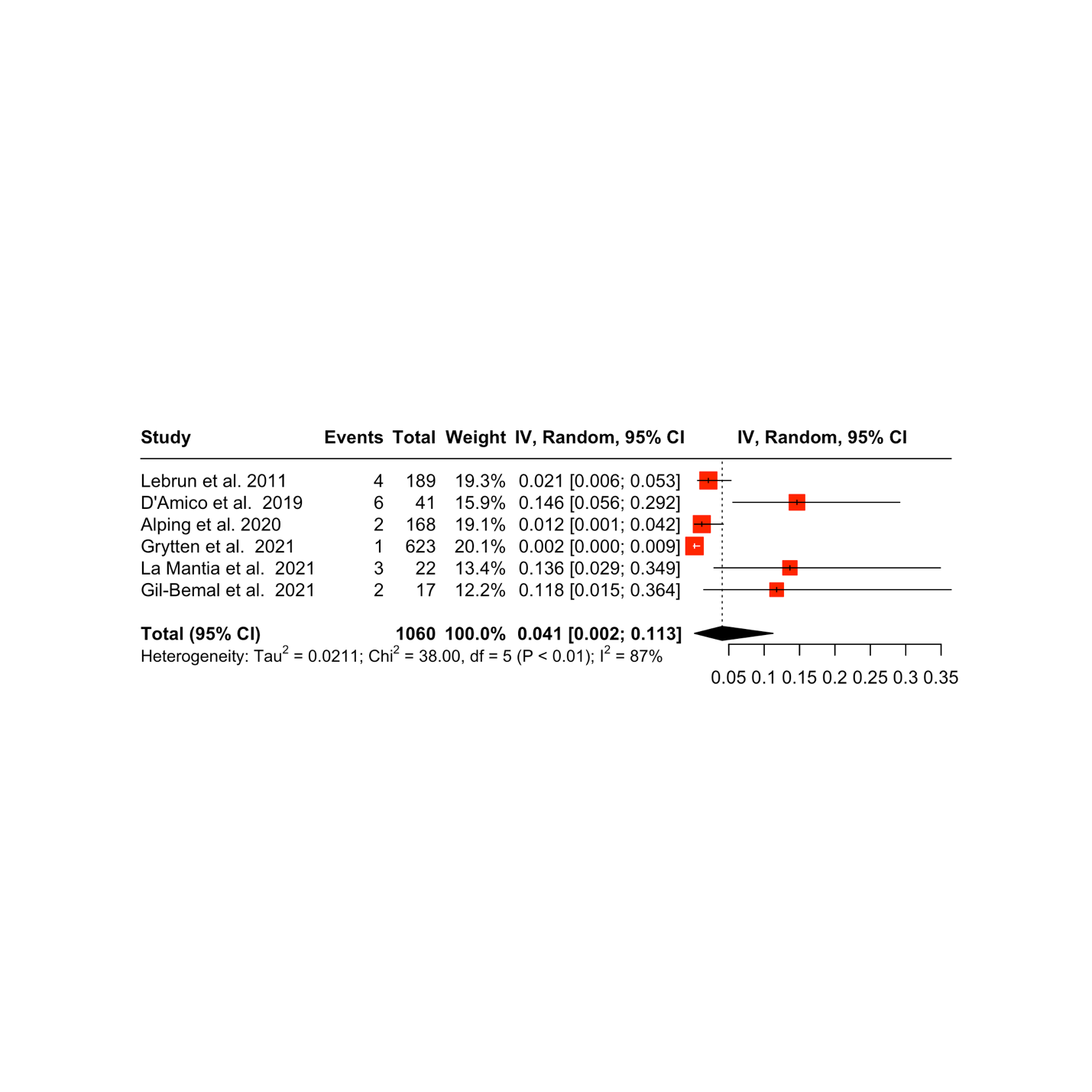
**

**Supplementary Figure 6: Forest plot: Pooled prevalence of Vesico-urethral cancer in PwMS under DMTs**

**
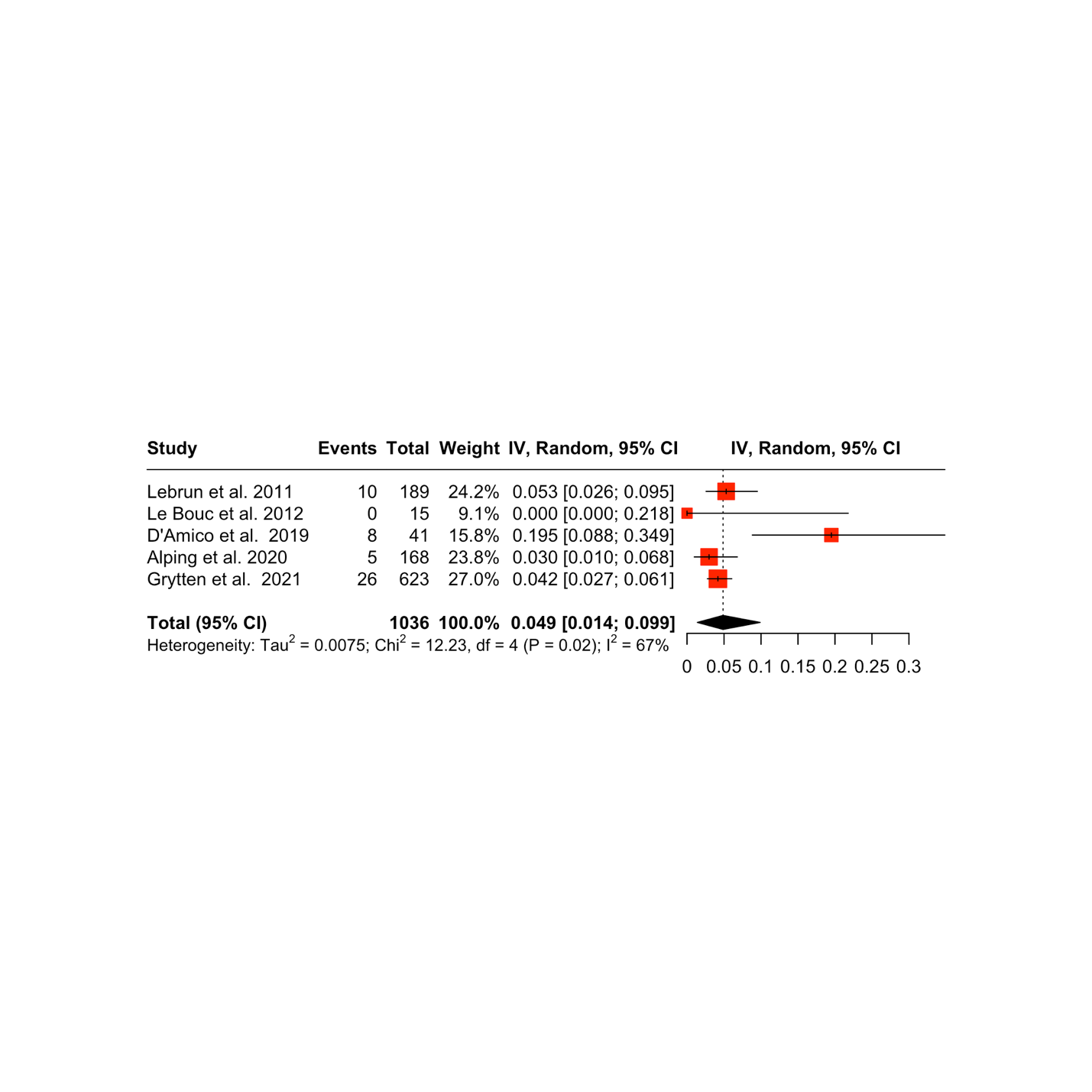
**

**Supplementary Figure 7: Forest plot: Pooled prevalence of Hematological cancer in PwMS under DMTs**

**
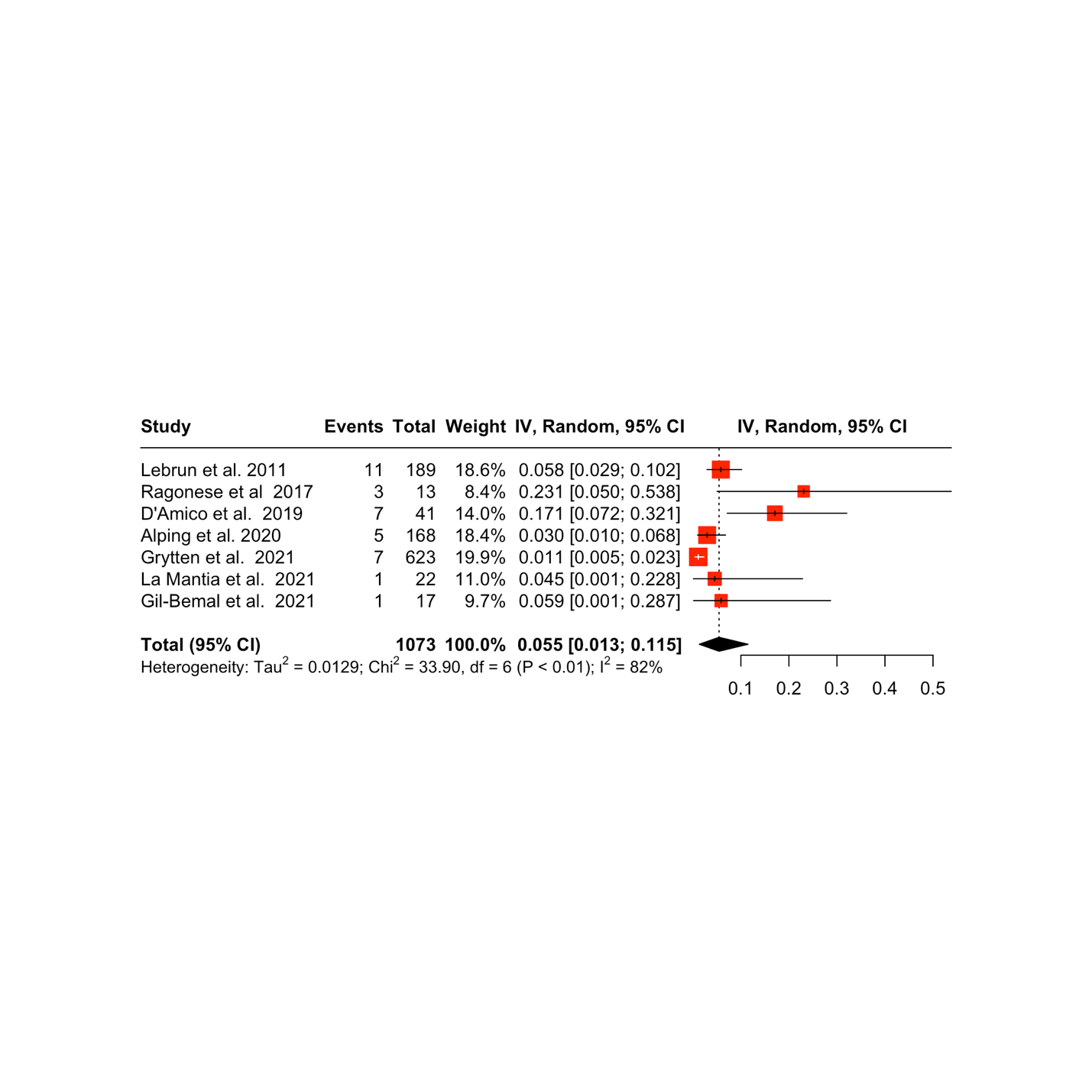
**

**Supplementary Figure 8: Forest plot: Pooled prevalence of Gynecological cancer in PwMS under DMTs**

**
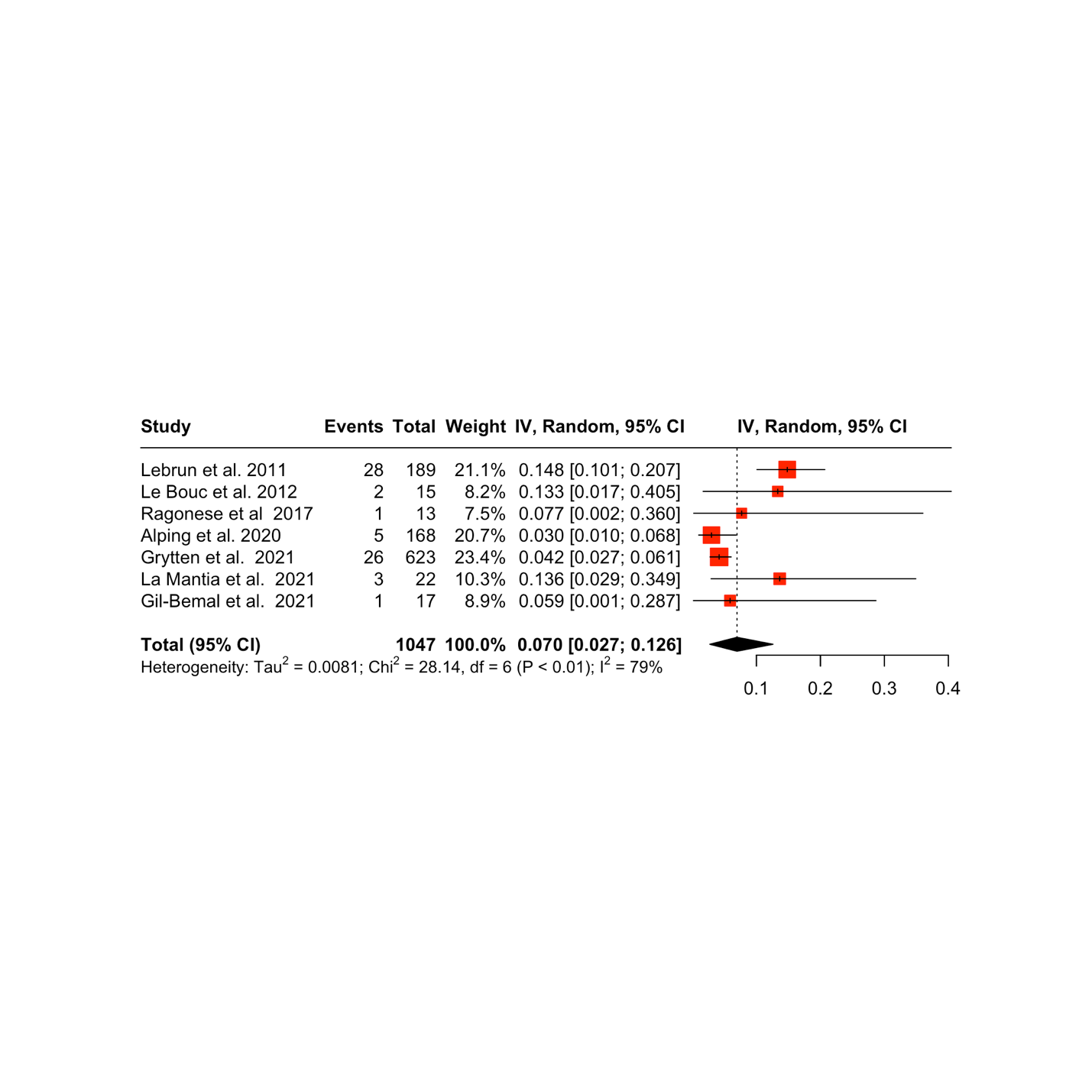
**

**Supplementary Figure 9: Forest plot: Pooled prevalence of Colorectal cancer in PwMS under DMTs**

**
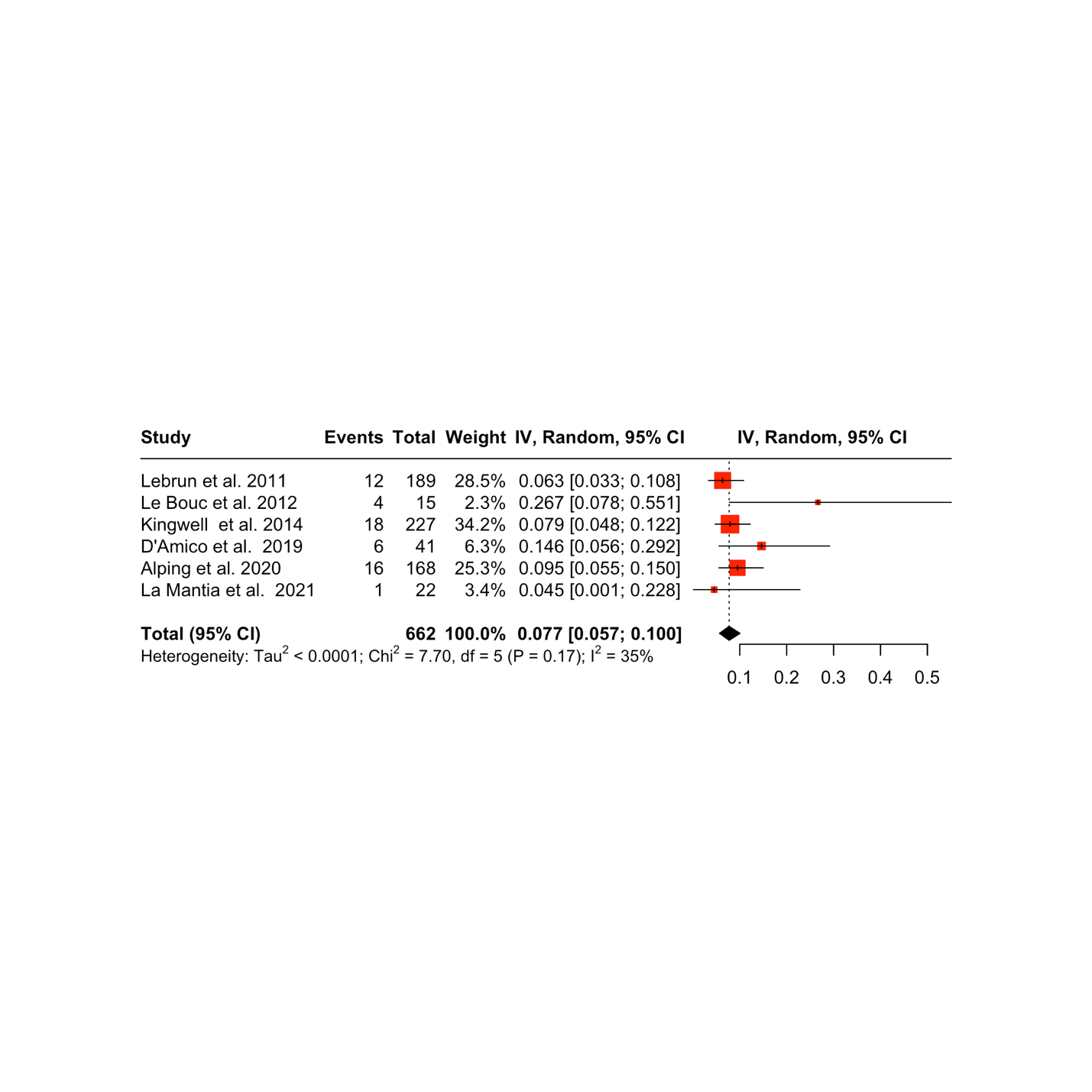
**

**Supplementary Figure 10: Forest plot: Pooled prevalence of Basal Cells Carcinomas in PwMS under DMTs**

**
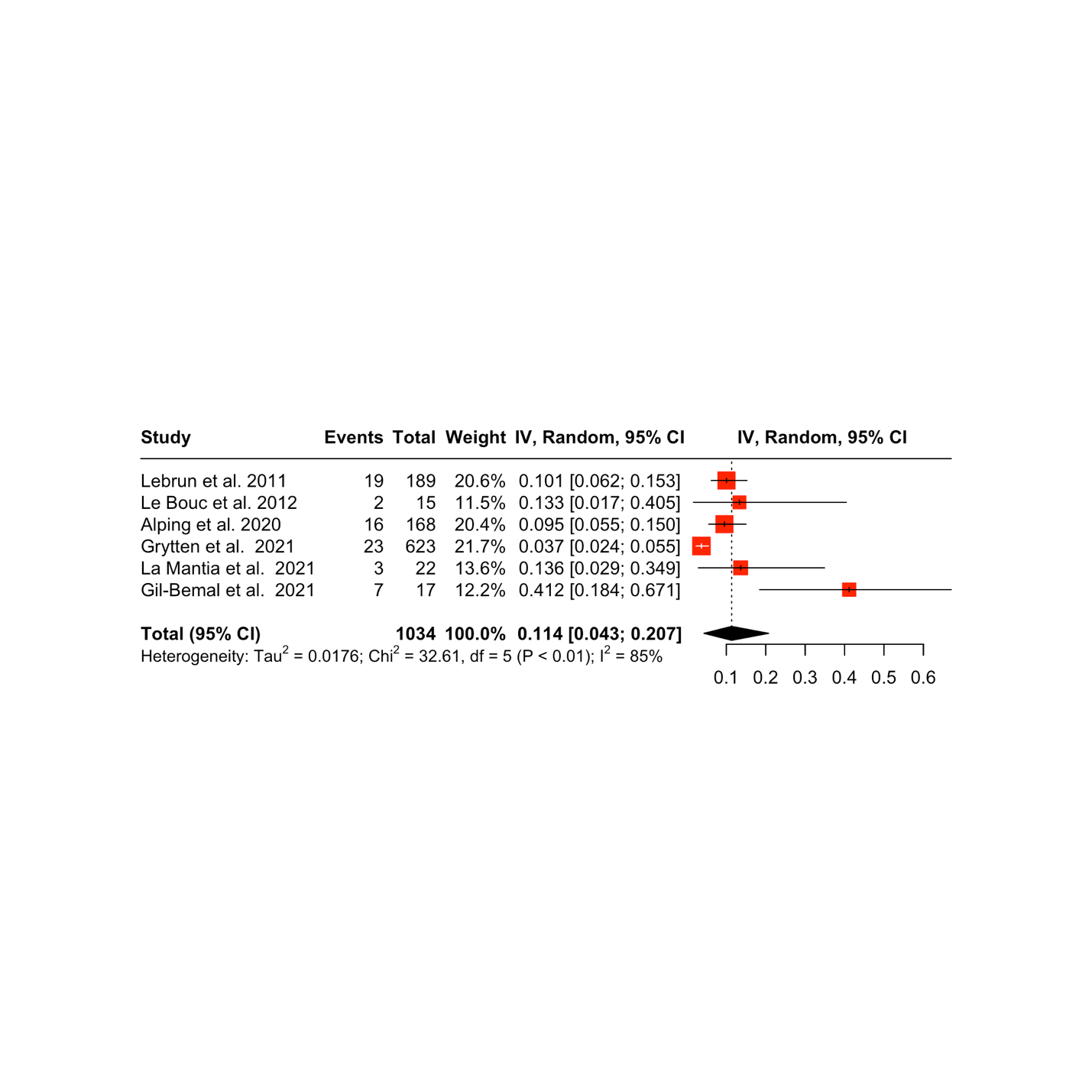
**

**Supplementary Figure 11: Forest plot: Pooled prevalence of Breast cancer in PwMS under DMTs**

**
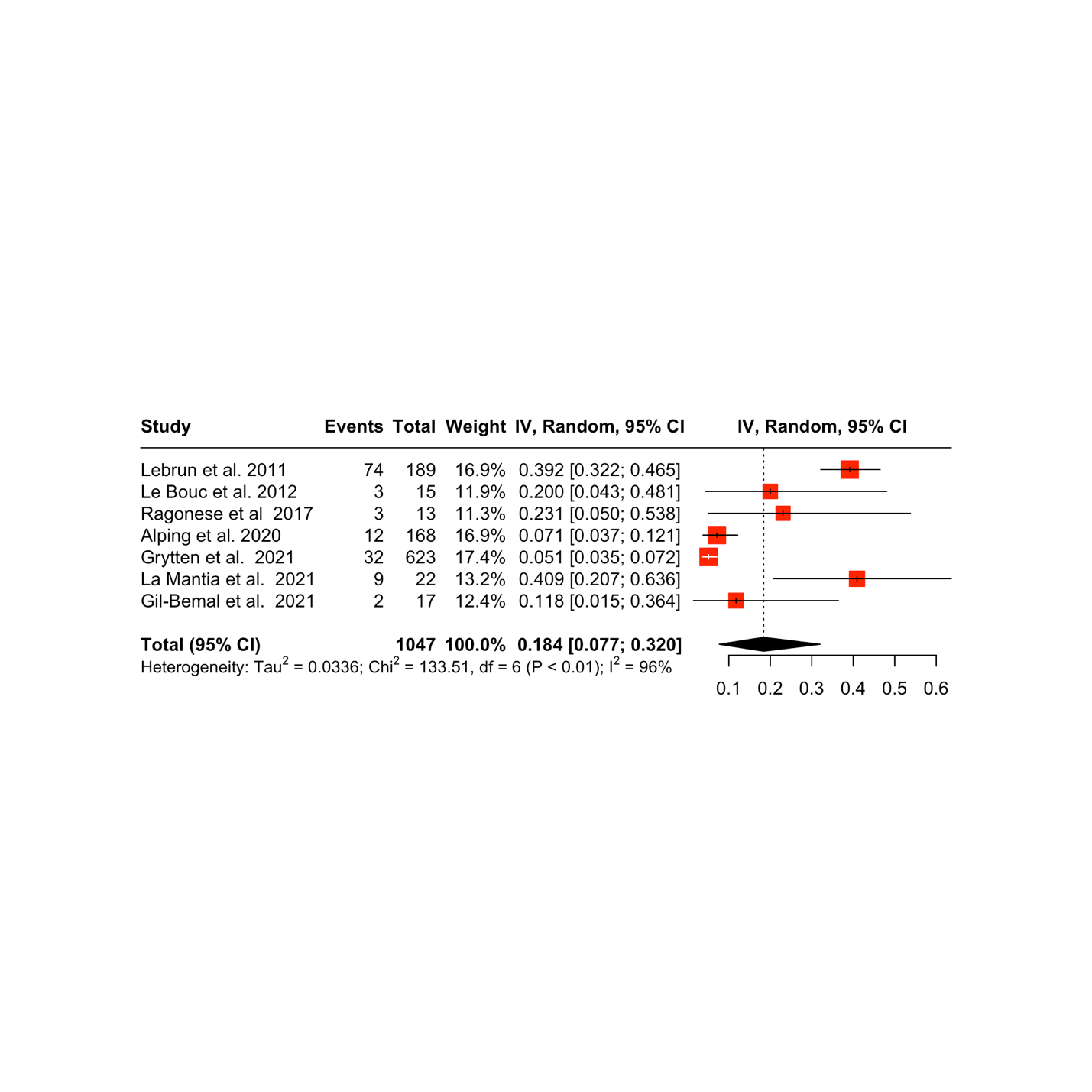
**

**Supplementary Figure 12: Funnel plot for publication bias**


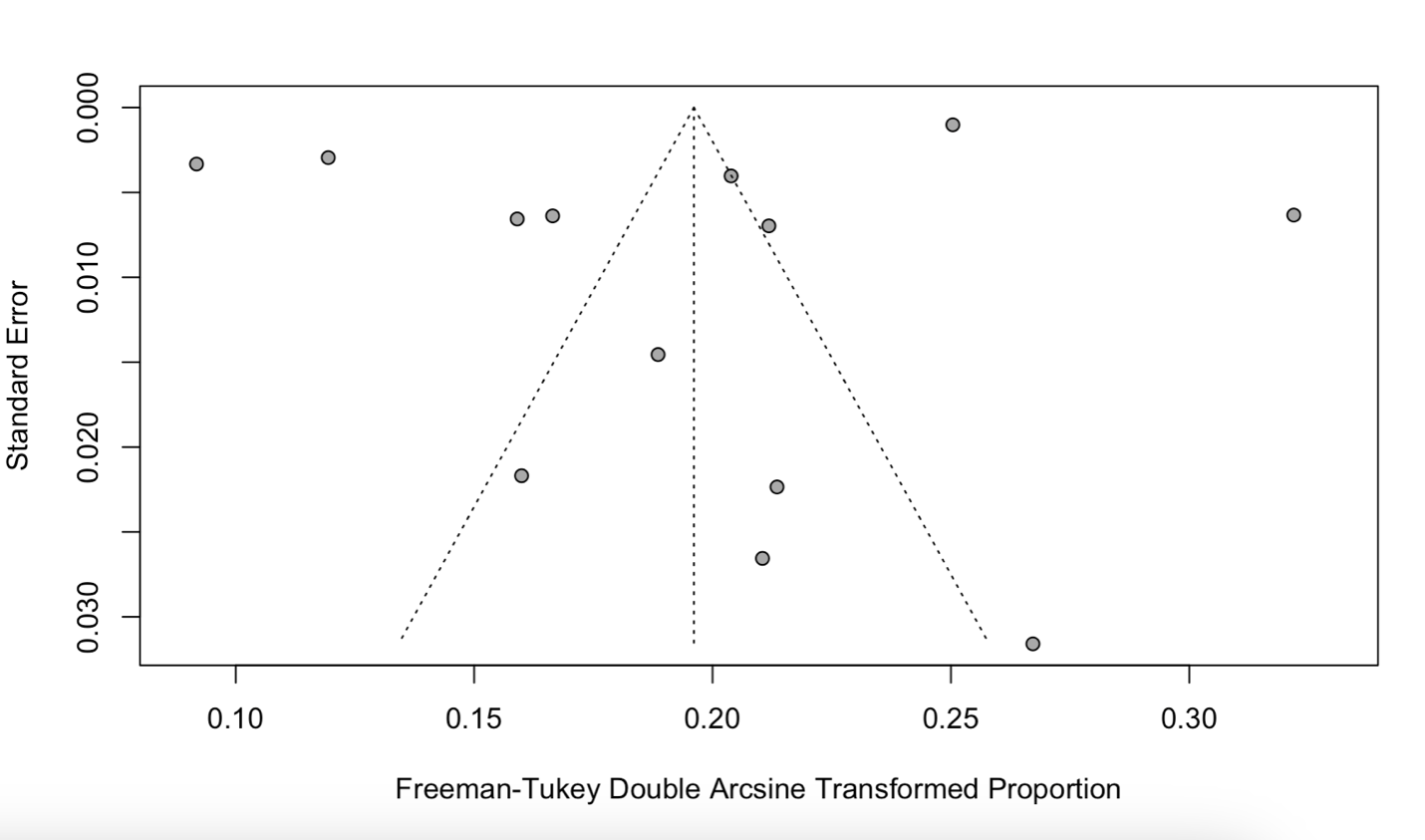


**Supplementary Figure 13: Prisma Checklist**

**
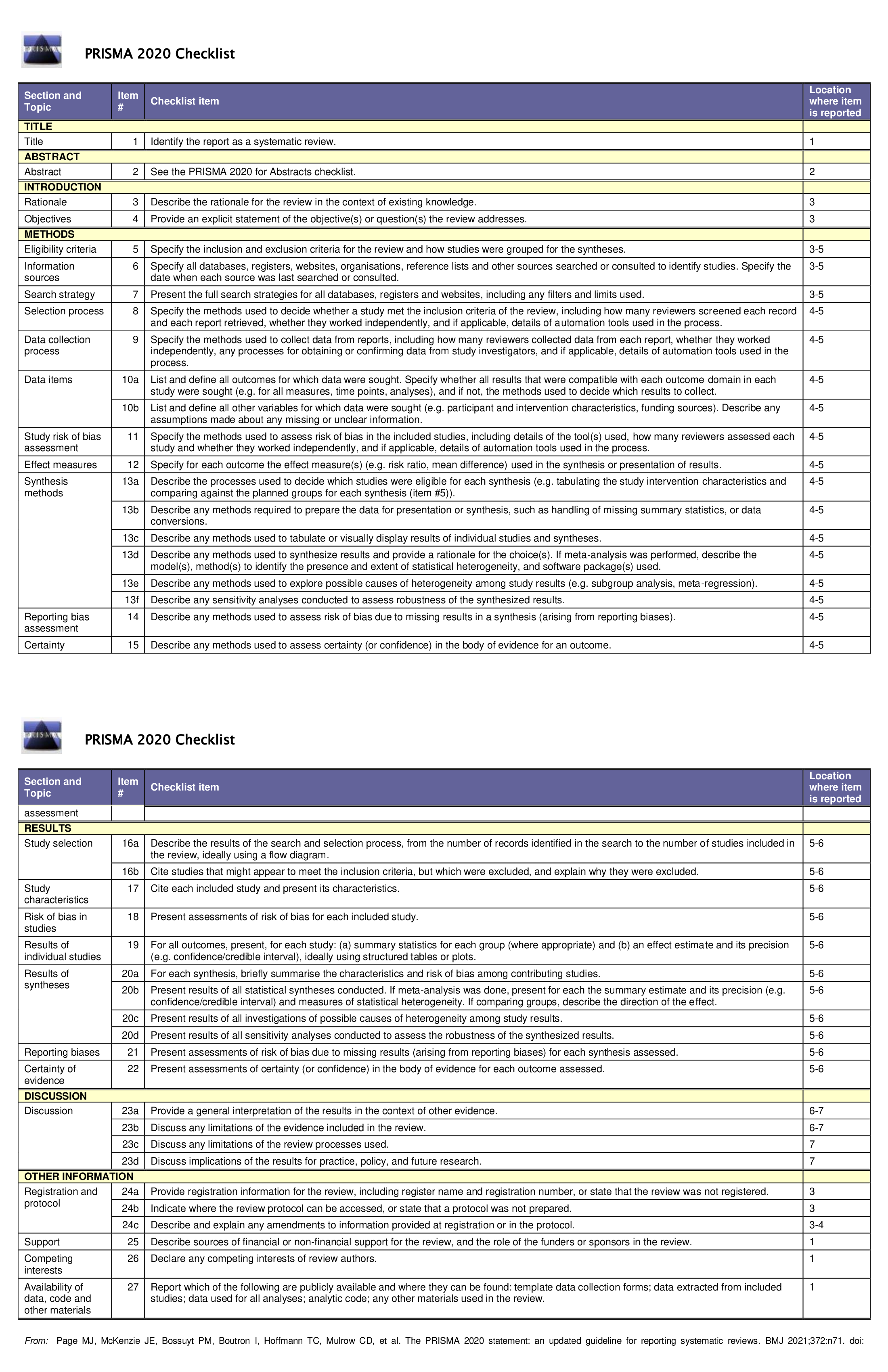
**

**4. References**

1. Giannopapas V, Palaiodimou L, Kitsos D, et al. The Prevalence of Diabetes Mellitus Type II (DMII) in the Multiple Sclerosis Population: A Systematic Review and Meta-Analysis. *J Clin Med* 2023; 12.

2. Stefanou MI, Palaiodimou L, Katsanos AH, et al. The effects of HMG-CoA reductase inhibitors on disease activity in multiple sclerosis: A systematic review and meta-analysis. *Mult Scler Relat Disord* 2022; 58: 103395.

3. Freeman MF and Tukey JW. Transformations related to the angular and the square root. *The Annals of Mathematical Statistics* 1950: 607-611.

4. Tsivgoulis G, Katsanos AH, Köhrmann M, et al. Duration of Implantable Cardiac Monitoring and Detection of Atrial Fibrillation in Ischemic Stroke Patients: A Systematic Review and Meta-Analysis. *J Stroke* 2019; 21: 302-311.

5. Borenstein M and Higgins JP. Meta-analysis and subgroups. *Prev Sci* 2013; 14: 134-143.

6. Egger M, Smith GD, Schneider M, et al. Bias in meta-analysis detected by a simple, graphical test. *Bmj* 1997; 315: 629-634.
